# Wheeze detection in real-world pediatric care: AI applied to smartphone lung auscultation

**DOI:** 10.1007/s00431-026-07036-9

**Published:** 2026-05-12

**Authors:** Inês Pais-Cunha, Diogo Pessoa, Maria Catarina Silva, Henrique Ferreira-Cardoso, Bruno Rocha, Rui Pedro Paiva, Paulo de Carvalho, João A. Fonseca, Inês Azevedo, Cristina Jácome

**Affiliations:** 1 Serviço de Pneumologia Pediátrica, UAG da Mulher e da Criança, ULS São João, Porto, Portugal; 2 RISE-Health, MEDCIDS-Department of Community Medicine, Information and Health Decision Sciences, Faculty of Medicine, University of Porto, Porto, Portugal, University of Porto, Porto, Portugal R. Dr. Plácido da Costa, 4200-450, Porto; 3 Centre for Informatics and Systems of the University of Coimbra, Department of Informatics Engineering, University of Coimbra, Coimbra, Portugal; 4 Serviço de Medicina Física E Reabilitação, ULS São João, Porto, Portugal; 5 Allergy Unit, Instituto CUF Porto E Hospital CUF Porto, Porto, Portugal; 6 Departamento de Ginecologia-Obstetrícia E Pediatria, Faculdade de Medicina da Universidade Do Porto, Porto, Portugal

**Keywords:** Pediatrics, Artificial intelligence, Smartphone, Respiratory sounds, Auscultation, Telemonitoring

## Abstract

Smartphone-based lung auscultation, combined with artificial intelligence (AI), may offer a promising alternative for pediatric respiratory telemonitoring. The aim of our study was to evaluate the performance of an AI model on wheeze detection from pediatric respiratory sounds recorded via smartphone. An observational cross-sectional study was conducted at the Pediatric Department of a Portuguese tertiary hospital, including children aged 0–17 years (pre-school, school-aged, and adolescent), with or without respiratory disease. Respiratory sounds were recorded at four locations using smartphone microphones and independently classified by at least 2 blinded annotators for quality and presence of wheezes. A hybrid convolutional–recurrent neural network (CNN + LSTM) trained on public electronic stethoscope databases was used to detect wheezes. AI model performance was assessed using positive predictive value, sensitivity, specificity, accuracy, and F1-score. A total of 217 children (59.9% male; median 10 [Q1-Q3 4.5–13] years) were included. From 2020 respiratory sound recordings, 1500 (74.3%) met quality criteria. Manual annotation identified 271 recordings with wheezes, whereas AI detected 217, most frequently in pre-school children (64.5% AI; 53.9% manual). The model achieved an overall accuracy of 87% (95% CI 86—89) and an F1-score of 61% (95% CI 56—66). The best accuracy was found in adolescents (92%, 95% CI 90—94) and the best global F1-score in pre-school children recordings (64%, 95% CI 58–71).

*Conclusion*: This study supports the feasibility of AI-assisted analysis of smartphone-recorded pediatric respiratory sounds in real-world settings. Future multicenter studies with larger datasets and model fine-tuning using smartphone recordings are expected to improve AI model performance and generalizability.

**Table:** 

**What is Known:** • *Continuous monitoring of pediatric respiratory diseases is essential to improve outcomes and reduce healthcare and family burden.* • *Smartphone lung auscultation is feasible in children but AI wheeze detection models have only been applied to recordings from electronic stethoscopes.*
**What is New:** • *First study applying an AI model to detect wheezes in smartphone-recorded lung sounds.* • *Quality recordings were obtained across all pediatric age groups and the AI model performance showed promising results, comparable to studies using electronic stethoscopes.*

**Supplementary Information:**

The online version contains supplementary material available at 10.1007/s00431-026-07036-9.

## Introduction

Respiratory diseases are among the leading causes of morbidity and healthcare burden in children worldwide [1]. It is essential to continuously monitor pediatric respiratory conditions, including asthma and less common chronic diseases such as cystic fibrosis, to detect early signs of poor disease control or deterioration and enable timely intervention [2, 3]. This is critical to improve clinical outcomes and reduce the burden on families and healthcare systems [4].

Mobile health (mHealth) has been evolving rapidly, offering new opportunities for remote monitoring and management of pediatric respiratory conditions, with the potential to improve disease control in both clinical and home settings [5–7]. Nevertheless, selecting the appropriate monitoring tools for this population remains challenging. Current technologies often rely on electronic questionnaires [8] and home spirometry [9, 10]. While these methods are valuable, they depend on patients’ active participation, which can hinder their application in younger children. Therefore, objective measures, such as digital lung auscultation which have shown potential [11] and do not depend on patient cooperation, are especially valuable in pediatric care at all ages [10].

Smartphone auscultation is a promising technology to integrate into remote monitoring systems. The widespread availability of smartphones equipped with high-quality microphones offers a practical, non-invasive, and scalable solution for recording respiratory sounds in clinical and home settings, thereby eliminating the need for additional devices, and enabling data collection through a single, familiar tool. Previous studies comparing conventional and smartphone lung auscultation have demonstrated real-world feasibility of the latter when performed by health professionals [12, 13] and caregivers [14]. Furthermore, caregivers found this technology highly acceptable [14]. These works demonstrated that smartphone lung auscultation was viable in recording adventitious respiratory sounds, such as wheezes, as validated by manual expert classification [12–14].

Wheezes are very common in children and usually indicate lower airway obstruction, a common finding in the most prevalent pediatric respiratory diseases [15, 16]. Although the detection of wheezes is a valuable aid in monitoring respiratory diseases, being correlated with radiographic findings and disease severity, its effectiveness depends on accurate identification, interpretation, and professional experience [17]. Due to the inherent subjectivity of manual classification, automated methods are needed [18].

A recent position paper by the European Academy of Allergy and Clinical Immunology emphasized the potential of artificial intelligence (AI) solutions in supporting pediatric clinical decision-making and disease monitoring [19]. In fact, this seems to be a powerful tool for interpreting complex physiological signals, including respiratory sounds [20–22]. Studies have evaluated the performance of AI algorithms on pediatric respiratory sounds captured by electronic stethoscopes, comparing the results to those of manual classification [22–24]. However, to date, no studies have explored the application of AI to respiratory sound recordings captured via smartphones. Given the potential synergy of combining these two technologies, our study aims to address this gap by evaluating the performance of an AI model on wheeze detection from pediatric respiratory sounds recorded via smartphone.

## Materials and methods

### Study design

An observational cross-sectional study was conducted with children at the Pediatric Department of the Local Health Unit of São João, a public tertiary hospital in Porto, Portugal. This study was reported in accordance with the recommendations of the STROBE (Strengthening the Reporting of Observational Studies in Epidemiology) statement [25]

### Participants

A convenience sample was recruited between September 2020 and June 2025. Children were included if aged 0 to 17 years-old (pre-school children 0–5 years, school-aged children 6–9 years, adolescents 10–17 years), with or without a respiratory disease (e.g., asthma, cystic fibrosis, and other respiratory diseases). Exclusion criteria included refusal to participate and children whose health status or condition prevented a harmless collection of respiratory sounds. Children and parents were invited to participate during a scheduled outpatient medical appointment, during their inpatient stay or at a visit to the emergency room.

### Data collection

Data on children’s sex, age, and height, as well as diagnostic group (asthma, cystic fibrosis, other respiratory disease, or no respiratory disease) were first registered in a paper case report form.

**Fig. 1:**
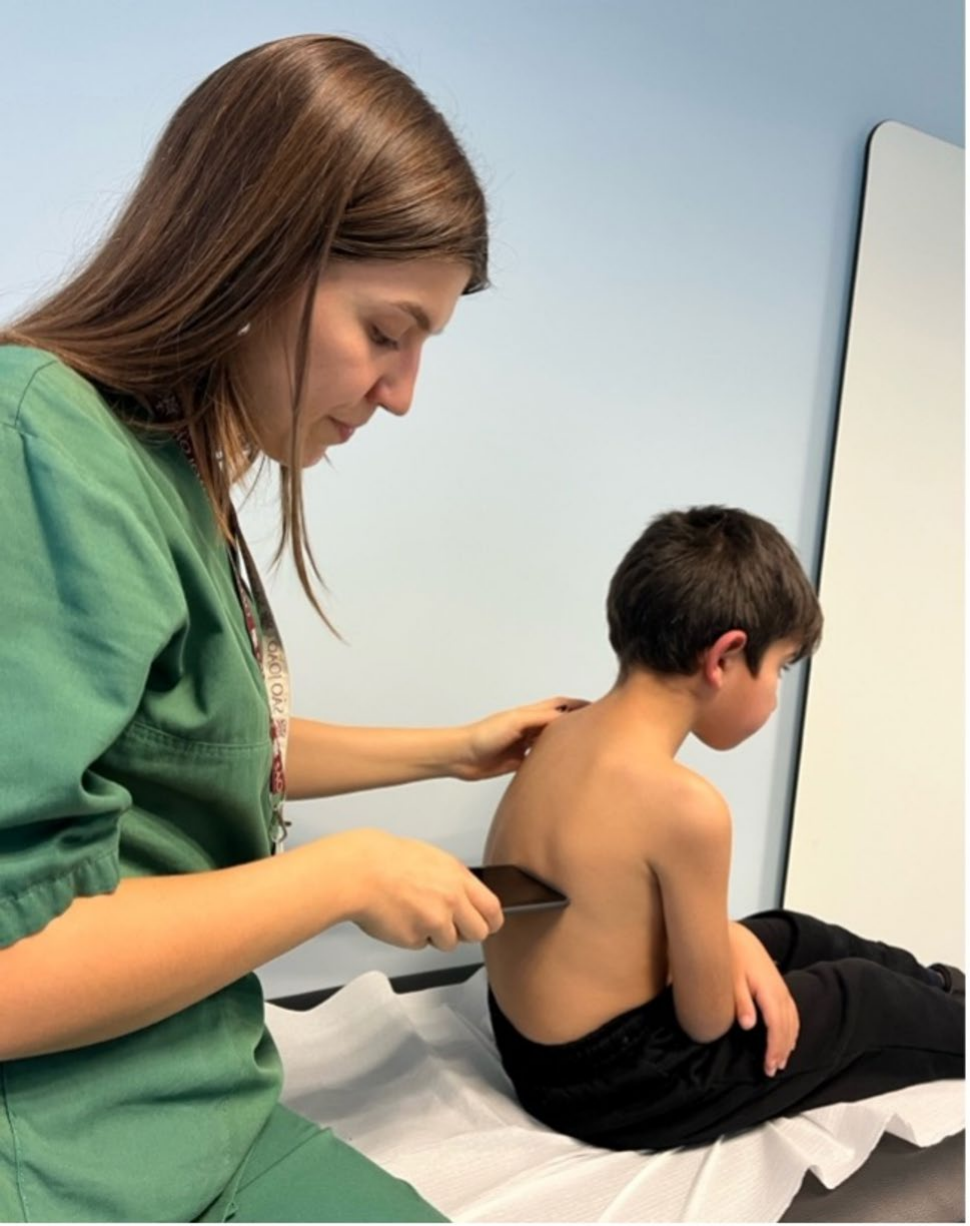
Lung auscultation with a smartphone microphone in clinic

Smartphone lung auscultation was performed using the lung auscultation feature of *InspirersKids* mobile app or a previous app version (AIRDOC) (Supplementary file 1, figure S1) [12, 26]. Respiratory sounds were recorded at four predefined locations: the three minimal recording sites recommended by the Computerized Respiratory Sound Analysis guidelines (trachea; right and left posterior inferior locations) [27] together with the additional right anterior site, included to capture potential adventitious sounds in the right middle lobe [28]. The smartphone was positioned at a ninety-degree angle, with the microphone pressed directly to the child’s skin, applying pressure to ensure that external noises were minimized (Fig. 1). Each auscultation location was recorded at least once in each location for 5–10 s.

A total of 15 smartphone models were used to collect the recordings (Supplementary file 1). Overall, 14 physicians participated in the acquisition process. Whenever consultation time allowed, parents performed additional recordings after receiving standardized instruction from the physicians, using the same smartphones as the physicians.

### Manual classification of respiratory sounds

All respiratory sound recordings were reviewed independently by at least two annotators. Five researchers were involved in the process: IPC, a pediatrician; HFC, CSS and MCS, medical students; and CJ, a physiotherapist/respiratory sound expert. During manual classification, the annotators knew only the patient’s identification number and the auscultation location and were blinded to all other piece of clinical information collected during the visit. In line with European Respiratory Society recommendations and previous work using predefined quality assurance criteria, we used prespecified study criteria to assess recording quality [29]. Recordings were judged dichotomously (yes/no) and were considered acceptable when they contained (1) minimal artefact, (2) an audible respiratory sound, whether normal or adventitious, suitable for analysis, and (3) clearly distinguishable respiratory phases. Given the short recording duration in this study (5–10 s), one complete respiratory cycle was considered sufficient to satisfy the latter criterion. Only recordings that met this quality threshold were retained for further analysis and classified as having the presence or absence of wheezes.

Disagreement between annotators regarding the quality of the recording or the presence/absence of wheezes was settled by consensus during online meetings and, when needed, an additional annotator was consulted (IA, pediatric pulmonologist/respiratory sound expert).

### AI classification of respiratory sounds

To automatically detect wheezes in smartphone recordings, an AI model, specifically an artificial neural network, was developed. The model had been trained on curated repositories of respiratory sounds recorded with electronic stethoscopes. Only sounds that had previously been manually classified as having quality were included in this analysis. The following subsections describe the data used for model training and its architecture.

#### Databases, pre-processing and data preparation

Currently, to the best of our knowledge, there are no public databases of smartphone-recorded respiratory sounds and corresponding annotations of respiratory adventitious sounds, specifically wheezes. Therefore, to develop the neural network model, several open-access databases of respiratory sounds recorded over electronic stethoscopes or similar devices were considered: the Respiratory Sound Database [30, 31]; the HF_Lung_V2 [32] and HF_Tracheal_V1 [33] datasets; and the SPRSound database [34]. The first one included recordings from children (mean age 4.8 ± 4.6 years) and adults; the second one only included adults and the third one included children up to 16 years. All databases considered included annotations for each individual wheezing event, with timestamps for its beginning and end. Before model training, raw audio waveforms were preprocessed and transformed into neural network inputs, including handcrafted audio features, mel-spectrograms, and Sobel-filtered mel-spectrograms. Description of databases, pre-processing and data preparation can be found in Supplementary file 2.

#### Model architecture and training

The model developed aimed to detect wheezing events within complete respiratory sound recordings, that is, determining the temporal onsets of such events. To perform event detection, we developed a hybrid deep learning model combining convolutional and recurrent modules (CNN + LSTM). Supplementary file 2 and figure S2 include information regarding the model architecture and the training pipeline.

#### Smartphone recordings classification and post-processing

The trained hybrid deep learning model (CNN + LSTM) was then used to automatically analyze smartphone-recorded respiratory sounds. Similar to the recordings used to train the neural network, the first step for the smartphone-recorded sounds was preprocessing, including normalization, filtering, feature extraction, and windowing. After decomposing each audio file into multiple 10—s windows, the model was used to detect wheezing events in each window. Whenever a recording had a duration shorter than 10-s, zero padding was applied to the raw audio to make the length of all recordings uniform. If the model detected a wheeze event in any of the windows of a given recording, that recording would be classified as containing wheezes. In addition to producing a binary classification for each recording (presence vs. absence of wheezes), the model also outputted the temporal localization of each detected wheezing event. An example of the model’s output is shown in a mel-spectrogram in Supplementary figure S3.

### Data analysis

Descriptive statistics were used to characterize participants regarding sex, age, age group (pre-school children, school-aged children, adolescents), height and diagnostic group (asthma, cystic fibrosis, other respiratory disease, and no respiratory disease). Continuous variables were expressed as medians and interquartile ranges [Q1-Q3]. Categorical variables were summarized using absolute and relative frequencies.

Pearson’s chi-squared test was used to compare categorical variables related to respiratory sound recordings (yes/no quality; presence/absence of wheezes) across age groups and auscultation locations, when there was no more than 20% of the expected values less than 5. When this assumption was not met, we used the Fisher’s exact test. When significant overall associations were found, post hoc tests were performed with Bonferroni correction for multiple comparisons.

Inter-rater agreement was evaluated separately for recording quality and presence of wheezes. Cohen’s kappa was used when the recording sound was classified by two-raters and Fleiss’ kappa when it was classified by three raters. Kappa values were interpreted using established cut-offs: < 0 = poor agreement, 0–0.20 = slight, 0.21–0.40 = fair, 0.41–0.60 = moderate, 0.61–0.80 = substantial, and 0.81–1.00 = almost perfect agreement [35].

AI model performance was evaluated using a confusion matrix. The metrics of positive predictive value (PPV), sensitivity, specificity, accuracy, and F1-score were included as evaluation parameters. PPV is the ratio of true positive predictions made from all positive predicted samples in the database. Sensitivity is the proportion of true positive cases correctly identified. Specificity is the proportion of true negative cases correctly identified. Accuracy is calculated as the proportion of correctly classified samples among all samples: (TP + TN)/(TP + TN + FP + FN), where TP, TN, FP, and FN indicate true positives, true negatives, false positives, and false negatives, respectively. The F1-score is the harmonic mean of PPV and sensitivity and provides a balanced measure of classification performance, 2 × (PPV × sensitivity)/(PPV + sensitivity). Statistical analysis was carried out using IBM SPSS Statistics (Version 29.0.2.0 Armonk, NY: IBM Corp). A *p*-value less than 0.05 was considered statistically significant.

## Results

### Children characteristics

A total of 217 children were included in the analysis. Most children were recruited during outpatient medical appointments (*n* = 175, 80.6%), followed by inpatient stays (*n* = 37; 17.1%) and emergency room visits (*n* = 5; 2.3%). The majority were male (*n* = 130; 59.9%). The median age was 10 [4.5–13] years. There were 53 (24.4%) participants with asthma, 15 (6.9%) with cystic fibrosis, 27 (12.4%) with other respiratory diseases, and 122 (56.2%) with no respiratory diseases. The participants’ characteristics are described in Table 1.

**Table 1:** Participants’ characteristics (*n* = 217)

Characteristics	Total	Pre-school children	School-aged children	Adolescents
Subjects, *n*	217	63	44	110
Male, *n* (%)	130 (59.9)	36 (57.1)	26 (59.1)	65 (59.1)
Age, median [Q1–Q3]	10 (4.5–13)	3 (0.9–4.0)	8 (7–9)	13 (11–16)
Height, median (Q1–Q3), cm	140.3 (110.5–158.9)	93 (77.5–104.5)	129 (120–133.5)	158 (148–164.125)
Primary diagnosis				
Asthma, *n* (%)	53 (24.4)	12 (19.1)	19 (43.2)	22 (0.2)
Cystic fibrosis, *n* (%)	15 (6.9)	2 (3.2)	2 (4.5)	11 (0.1)
Other respiratory disease, *n* (%)	27 (12.4)	24 (38.1)	0 (0)	3 (0.3)
No respiratory disease, *n* (%)	122 (56.2)	25 (39.6)	23 (52.3)	74 (67.3)

Q1, first quartile; Q3, third quartile

### Manual classification and quality of respiratory sounds

A total of 2020 respiratory sounds were collected, of which 346 (17%) were collected by parents. Inter-rater agreement on manual classification ranged from 0.69 to 0.80 when using Cohen’s Kappa and 0.58 to 0.70 when using Fleiss kappa (Supplementary file 3).

Of these, 1500 (74.3%) sounds were considered as having quality. These 1500 sounds corresponded to 209 patients, whose characteristics are detailed in Supplementary table S1. There was no significant difference in the proportion of quality of sounds collected by parents and physicians (73.7% vs 74.4%, *p* = 0.421).

Regarding age group, quality recordings included 394 (67.2%) from pre-school children, 299 (73.8%) from school-aged children, and 807 (78.4%) from adolescents. Regarding recording quality by location, 361 (74.7%) were from the right anterior, 341 (69.6%) from the right posterior inferior, 397 (72.3%) from the left posterior inferior, and 401 (80.5%) from the trachea (Supplementary figure S4).

### AI model performance

**Fig. 2:**
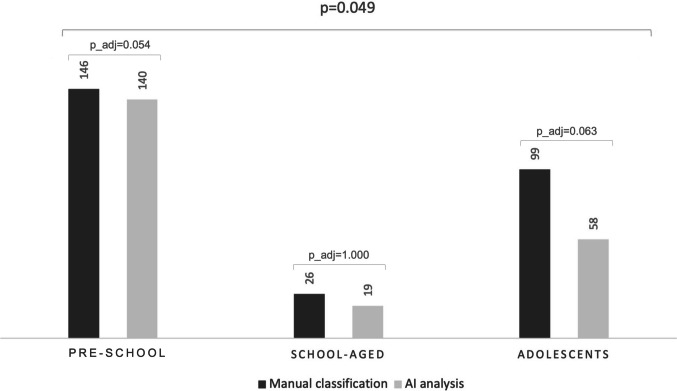
Detection of wheezes by manual classification (*n* = 271) and AI model (*n* = 217) across age groups. The top *p*-value refers to the overall Pearson’s chi-square test (2 × 3); p_adj indicates Bonferroni-adjusted *p*-values (m = 3) for post hoc comparisons between methods within each age group

**Fig. 3:**
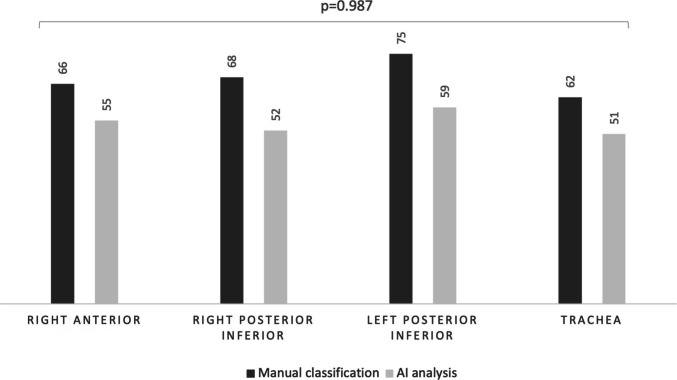
Detection of wheezes by manual classification (*n* = 271) and AI model (*n* = 217) by auscultation location (right anterior location, right posterior inferior, left posterior inferior trachea). The top *p*-value refers to the overall Pearson’s chi-square test (2 × 3)

Manual classification identified 271 recordings containing wheezes, while automated analysis detected 217. In both methods, the majority of these sounds occurred in pre-school children (automated: 64.5%; manual: 53.9%). The overall age-group distribution differed between methods (*p* = 0.049), but no post hoc comparison remained significant after Bonferroni adjustment (pre-school p_adj = 0.054; school-aged p_adj = 1.000; adolescents p_adj = 0.063) (Fig. 2). Regarding auscultation locations, wheezes detection was similar between the two methods (*p* = 0.987) (Fig. 3).

**Fig. 4:**
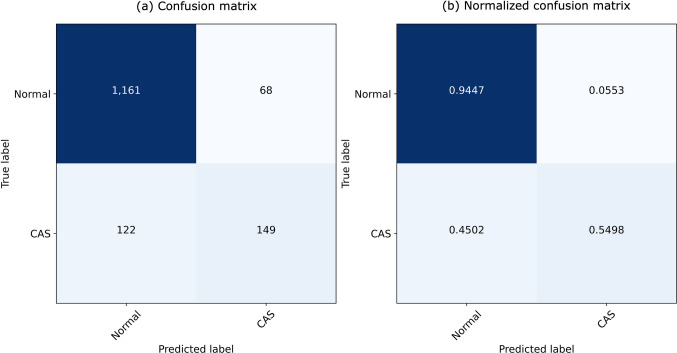
**(a)** Confusion matrix and (**b)** normalized confusion matrix of overall IA model performance

Model performance is summarized in Table 2, and overall performance is represented in a confusion matrix in Fig. 4. The AI model achieved an overall accuracy of 87% (95% CI 86–89), with the best performance observed in the adolescents’ recordings (92%, 95% CI 90–94) and at the left posterior inferior location (91%, 95% CI 88–93). The global F1-score was 61% (95% CI 56–66), with the best performance observed in pre-school children (64%, 95% CI 58–71) and at the left posterior inferior location (69%, 95% CI 61–78). Supplementary figures S5 and S6 represent confusion matrices of IA model performance according to age group and auscultation locations, respectively.

**Table 2:** Analysis of artificial intelligence (AI) model performance on all respiratory sounds, by age group and auscultation location

Respiratory sounds	PPV (%, 95% CI)	Sensitivity (%, 95% CI)	Specificity (%, 95% CI)	Accuracy (%, 95% CI)	F1-score (%, 95% CI)
All	69 (62–74)	55 (49–61)	94 (93–96)	87 (86–89)	61 (56–66)
Age group					
Pre-school children	66 (58–73)	63 (55–70)	81 (75–84)	74 (70–78)	64 (58–71)
School-aged children	47 (27–68)	35 (19–54)	96 (92–98)	91(87–94)	40 (22–58)
Adolescents	83 (71–90)	48 (39–58)	99 (97–99)	92 (90–94)	61 (52–70)
Auscultation location					
Right anterior	71 (58–81)	59 (47–70)	95 (91–97)	88 (84–91)	64 (54–74)
Right posterior inferior	66 (55–76)	69 (57–79)	91 (87–94)	88 (84–91)	68 (59–77)
Left posterior inferior	68 (57–77)	71 (60–80)	92 (89–95)	91 (88–93)	69 (61–78)
Trachea	42 (29–55)	35 (25–48)	91 (87–93)	82 (78–86)	38 (27–50)

*CI* confidence interval, *PPV* positive predictive value

## Discussion

To our knowledge, this is the first study to test an AI classification model on pediatric respiratory sounds recordings collected with smartphone microphones. We highlight three key findings of our study: quality recordings were obtained across all age groups in a real-world setting; our AI model performed well when applied to smartphone respiratory sound recordings and its best performance was achieved at the left posterior inferior chest location.

Deep learning models are well suited for respiratory sound analysis as they can automatically learn relevant signal characteristics and identify complex visual and time–frequency patterns, such as a wheeze, in spectrogram-based representations [36, 37]. Our AI model used mel-spectrograms, one of the most commonly reported feature extraction techniques in pediatric lung sound analysis, together with a CNN-based hybrid architecture, an approach widely described in the literature [37]. In addition to detecting wheezing recordings as a binary outcome, the model’s temporal localization enabled predicted events to be linked directly to specific segments of the audio signal, providing an intuitive form of interpretability and supporting practical validation of the results. Overall, the model’s performance results fall within the range reported in recent systematic reviews. For pediatric asthma, these reviews report F1-scores between 66.4 and 87.2% and accuracy values of 57.7–97.7% [38]. For pediatric respiratory sounds more broadly, reported F1-scores range between 32.96 and 95.2% and accuracy between 69.72 and 100% [37]. The substantial heterogeneity in study populations, annotation protocols, and evaluation metrics across studies limits the ability to make direct comparisons of reported performance. Although we had a substantial total number of sounds, larger than that of most cohorts, the proportion of adventitious sounds was lower than in studies with higher F1-scores [21, 39]. The lower representation of adventitious sounds in our database led to class imbalance, making wheezing events harder to detect and consequently reducing F1-scores. A greater proportion of adventitious sounds would probably improve model sensitivity for adventitious sound detection. Nevertheless, the interpretation of these metrics is context dependent. In our study, the moderate performance observed may still be acceptable for wheeze monitoring as part of a broader clinical assessment, similarly to other pediatric AI models developed to support prognostic stratification and personalized clinical decision-making [40]. However, it would be less suitable for diagnostic purposes [41].

We acknowledge that AI performance may have been influenced by the fact that the model was not trained on smartphone-recorded sounds. Although auscultation with electronic stethoscopes and smartphone microphones has been shown to be comparable [13], the acoustic characteristics of these devices differ, including their sensitivity and frequency response. These differences may alter the spectral representation of respiratory sounds and contribute to performance variability [42].

From an anatomical perspective, performance was better in the left posterior inferior lung area. This is consistent with the fact that posterior basal areas are closer to the pulmonary parenchyma. These locations are also less affected by interference from bone and muscle structures, which favors sound transmission [37, 43]. However, although previous studies have reported better performance when auscultating over the trachea in children, which has been attributed to lower attenuation of chest wall sounds [44], our model performed worse at this location. This may be related to pediatric anatomy—the shorter trachea and smaller surface area, especially in younger children, make smartphone positioning more difficult. In turn, this could potentially introduce artifacts and affect the interpretability of respiratory sound recordings. In our sample, differences in performance across age groups appear to be largely driven by the proportion of adventitious sounds. These were more prevalent among pre-school children, leading to a more balanced distribution of classes and consequently higher F1-scores. In adolescents, adventitious sounds were much rarer, creating a substantial class imbalance that lowered the F1-score despite higher overall accuracy.

We need to consider that our study was conducted entirely in real-world settings and all recordings were obtained in hospital environments. Overall, the outcome was positive, with 74% of the collected sounds meeting the required quality standards. Our approach builds on previous work by Kang et al. in cardiac auscultation, which showed that smartphone microphones can feasibly record heart sounds for AI-based analysis, despite acquisition challenges [45]. We found no significant differences between recordings collected by parents and physicians. However, we need to consider that the parent recordings were performed after physician instruction and under clinical supervision. While these findings support the feasibility of involving parents in smartphone lung auscultation, future studies should evaluate this approach in unsupervised settings to determine its applicability for home monitoring. Although recordings were obtained according to a standardized protocol, data collection took place in routine healthcare settings, where environmental conditions were not fully controlled, reflecting everyday clinical practice. As a result, recordings remained susceptible to background noise and movement-related artefacts, including speech and patient motion, which could potentially impact quality classification. This may partly explain the lower proportion of quality recordings among pre-school children, who tend to cooperate less during lung auscultation. As mentioned before, we observed a relatively low prevalence of wheezes (approximately 20%). This likely reflects the clinical profile of our cohort, as most children did not have respiratory disease and many recordings were obtained during scheduled outpatient appointments, when patients are generally more stable. Compared to other studies on digital lung auscultation, our findings are in line with those of Habukawa et al. in outpatient children with mild asthma (30%) [46] and notably lower than those reported by McCollum et al. in hospitalized children with severe pneumonia (62%) [47].

This study has some limitations that must be acknowledged. One limitation relates to the use of different smartphones and the involvement of multiple individuals in data collection, which may have introduced variability in the recordings. Even though this heterogeneity reflects real-world conditions and may support the generalizability of our findings, differences in audio recording characteristics across devices may also have affected sound quality and signal consistency. Additionally, although recordings were performed according to a standardized protocol, the absence of automatic real-time feedback regarding smartphone positioning and sound recording may also have impacted the quality of recordings. Incorporating real-time auditory or visual guidance into future technologies could enhance both the quality and usability of recordings. Another limitation concerns the manual classification of respiratory sounds, which was performed by five healthcare professionals, although one annotator was common to all analyses. Future studies employing a consistent panel of annotators and a standardized classification method, such as audiovisual recordings [48, 49], could help reduce inter-observer variability and improve comparability across datasets. Finally, reliance on at least one full, distinguishable respiratory phase within brief 5–10 s recordings was necessary to accommodate short audio samples. However, this approach may be insufficient for capturing respiratory variability and episodic events, potentially introducing bias in sound classification.

Despite the limitations of our study, we provide a valuable dataset of real-world pediatric respiratory sounds recorded with smartphone microphones. To further improve and validate the AI model, future research should expand the dataset with a larger number of smartphone-recorded sounds. This would allow for more effective training and independent testing, a standard approach we could not implement in this study given the relatively small sample size. Improving the model’s performance would also likely involve retraining it with a larger set of adventitious sounds and a more balanced representation of different age groups. Furthermore, as respiratory sound characteristics differ significantly with age, the model could be adapted for each demographic. Independent testing and customization for each target age group would make the model more suitable for future clinical applications.

With continued improvement and real-world testing, smartphone lung auscultation has the potential to serve as a supportive tool for telemonitoring respiratory diseases in children. In the long term, it could be used by children and their caregivers to collect longitudinal auscultation data, which could then be combined with other clinical information to inform clinical decision-support systems, ultimately supporting more accessible and scalable respiratory assessment. More broadly, the growing use of smartphones may enable larger and more diverse respiratory sound datasets, helping to make lung auscultation more objective and standardized, with potential value for both clinical practice and teaching [50].

In conclusion, our research suggests that combining smartphone lung auscultation with AI technology is a promising approach for analyzing children’s respiratory sounds. While further optimization and validation are necessary, our results support the potential value of this method in clinical practice.

## Supplementary Information

Below is the link to the electronic supplementary material.

ESM 1 Supplementary Material 1 (DOCX 1.94 MB)

## Data Availability

No datasets were generated or analysed during the current study.
