# Supplementary material for: Wheeze detection in real-world pediatric care: AI applied to smartphone lung auscultation
Source: Eur J Pediatr. 2026 May 12;185(6):383. doi: 10.1007/s00431-026-07036-9 (PMC13167856; doi:10.1007/s00431-026-07036-9)
Supplement: Supplementary file 1 — Supplementary Material 1 (DOCX 1.94 MB) [file 431_2026_7036_MOESM1_ESM.docx]

**Contents**

[Supplementary file 1 – AIRDOC and InspirersKids app description 2](#_Toc225445720)

[Supplementary figure S1 3](#_Toc225445721)

[Supplementary file 2 – Database description; pre-processing and data preparation; model preparation and training 4](#_Toc225445722)

[Supplementary figure S2 7](#_Toc225445723)

[Supplementary figure S3 8](#_Toc225445724)

[Supplementary file 3 - Inter-rater agreement on manual classification 9](#_Toc225445725)

[Supplementary table S1 10](#_Toc225445726)

[Supplementary figure S4 11](#_Toc225445727)

[Supplementary figure S5 12](#_Toc225445728)

[Supplementary figure S6 14](#_Toc225445729)

[References 16](#_Toc225445730)

# Supplementary file 1 – AIRDOC and InspirersKids app description

The AIRDOC app is a mobile app which aims to monitor individuals with chronic respiratory diseases, using the smartphone and its integrated sensors. The app enables the recording of sounds, from lung auscultation or forced expiratory maneuvers, as well as to complete validated questionnaires.[1] The *InspirersKids* app aims to assist telemonitoring of children with respiratory diseases. This mobile app was created based on the AIRDOC app and adapted to children.[1] These apps are available on iOS and Android. In this study, only the lung auscultation feature was used. This feature enables the recording of respiratory sounds in the 7 locations recommended by CORSA guidelines[2] or other locations, with an adjustable duration. Each recording includes automatic information of the location and duration. It is also possible to write additional information in a note field. The current versions do not include any signal processing of the sound files.

15 smartphone models were used to record respiratory sounds: iPhone 6s (iOS), iPhone 7 (iOS), iPhone 8 (iOS), iPhone XR (iOS), iPhone 11 (iOS), iPhone 12 Pro (iOS), iPhone 13 (iOS), iPhone 14 (iOS), iPhone 15 (iOS), iPhone 15 Pro (iOS), Xiaomi M9 Pro (Android), Xiaomi Note 8 (Android), Huawei P10 Lite (Android), Huawei Nova 5T (Android), and One Plus 7 Pro (Android).

The apps were used offline. Whenever an internet connection was available, the data was automatically transferred to a secure server at the Faculdade de Medicina da Universidade do Porto, from which the recordings could be accessed by authorized researchers.

Supplementary figure S1


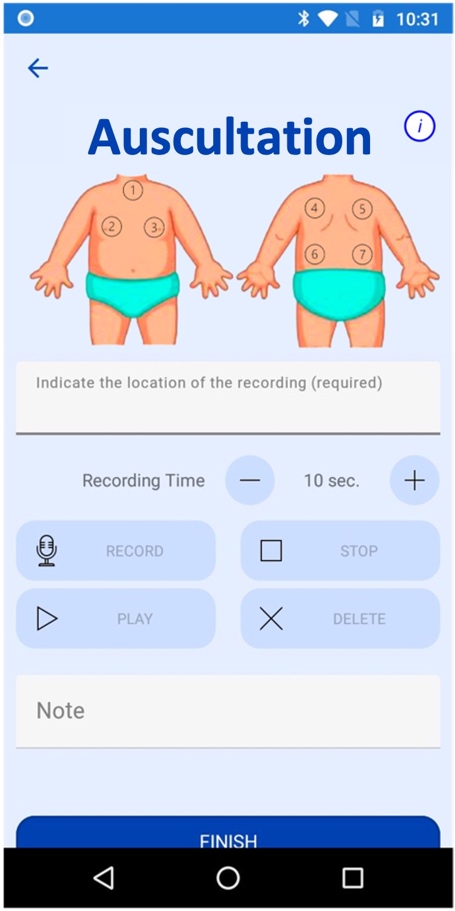


Supplementary Figure S1– *InspirersKids* lung auscultation feature screen layout.

# Supplementary file 2 – Database description; pre-processing and data preparation; model preparation and training

*Database description*

The Respiratory Sound Database (RSD)[3; 4] is widely adopted to develop methods for automated respiratory sound processing and analysis. It consists of 5.5 hours of annotated respiratory sounds, comprising 920 audio samples from 126 patients with multiple respiratory conditions. The recordings yield 6898 respiratory cycles, of which 1864 contain crackles, 886 contain wheezes, and 506 contain both. The recordings were collected from multiple chest locations, using various recording devices, namely electronic stethoscopes (3M Littmann 3200, Welch Allyn Master Elite Plus - Meditron, 3M Littmann Classic II SE) and digital microphones (C 417 PP, AKG Acoustics).

HF_Lung_V2[5] is an open-access database established to serve as a benchmark for both classification and segmentation tasks in respiratory sound analysis. With 13 957 15-second recordings totaling approximately 58 hours and 9 minutes of audio, it is the largest publicly available repository of respiratory sounds. In total, the database has recordings from 300 participants, collected at a respiratory care center and at the Far Eastern Memorial Hospital in Taipei, Taiwan. Regarding the annotation events, it contains 49,373 inhalation (I) events, 24 552 exhalation, 21 558 continuous adventitious respiratory sound (C) events, and 19 617 discontinuous adventitious respiratory sound (D) events.

The SPRSound database[6] consists of 2683 recordings and 9089 respiratory sound events collected from 292 pediatric participants. The participants were recruited from the Shanghai Children’s Medical Center. The age of participants ranges from 0.2 to 16.2 years, with a mean age of 5.4 years. This dataset includes respiratory conditions such as asthma, bronchitis, and pneumonia (severe and non-severe), as well as other respiratory diseases and healthy children. Respiratory sounds were recorded using an electronic stethoscope (Yunting model II) at a sampling rate of 8 kHz and a 16-bit quantization resolution.

*Pre-processing and data preparation*

Prior to model training, a comprehensive preprocessing and feature-extraction pipeline was implemented to transform raw audio waveforms into meaningful numerical representations suitable for neural network input, namely handcrafted audio features, mel-spectrograms, and Sobel-filtered mel-spectrograms.

All recordings were first normalized to -1 to 1, resampled to 4kHz (the minimum sampling rate across all recordings from the multiple databases), and filtered with a high-pass Butterworth filter at 80Hz. Then, we have extracted multiple features, typically used in the context of respiratory sound analysis[4; 7], as well as the mel-spectrogram computation (time-frequency representation).[7] The extracted features were the following: root mean square energy (RMS), zero-crossing rate (ZCR), spectral centroid, spectral bandwidth, spectral flatness, spectral roll-off, spectral flux, energy summation by band, and Mel-Frequency Cepstral Coefficients (MFCCs) with their respective deltas. In total, 77 features were extracted.

To compute the mel-spectrogram, we used a 64-ms Blackman–Harris window with 75% overlap and 64 mel-bandpass filters. Sobel filters[8] were then applied to the TF representations in the horizontal direction to emphasize edges and frequency transitions.

After feature extraction and mel-spectrogram computation, the data for each entire recording were normalized using Z-score normalization.

Binary activation vectors aligned with each frame of the audio signal's representation were created, allowing the neural network to learn the timing and duration of wheezing events. Such vectors encode the presence or absence of a wheezing event at a given temporal instant, based on expert annotations.

Given differences in audio file length across databases, we used a sliding window approach to divide the complete audio signals into multiple fixed-sized windows.[9] In particular, we have decomposed all windows into multiple 10-second windows, with 50% overlap. The windowing process was also applied to the binary activation vectors.

*Model architecture and training*

While the convolutional block automatically extracted features from the time–frequency representations and respiratory sounds (i.e., feature learning), the recurrent module learned their temporal dependencies and modulated them over time.

As stated in the ‘Pre-processing and data preparation’ subsection, the model receives three inputs: a mel-spectrogram, a Sobel-filtered version of the mel-spectrogram, and a set of features. The two time-frequency representations are concatenated to form a unified spectral branch, which is then permuted to ensure proper alignment of the time and frequency dimensions before convolutional processing. Similarly, the handcrafted feature input is permuted to match the temporal axis. Both spectral and feature branches are individually processed through convolutional layers with 32 filters and a kernel size of (5, 5), each followed by ReLU activations, batch normalization, and spatial dropout with a 10% dropout rate. The two processed branches are later concatenated to form a representation that integrates spectral and feature-level information. Two additional convolutional blocks follow the concatenated output. The first block uses 64 filters and is followed by a ReLU activation function, a batch normalization, and a spatial dropout layer with a 10% rate. The second block utilizes 128 filters and is structured similarly to the previous block.

After feature extraction, a global max pooling is applied along the feature dimension, producing a condensed time-aligned representation. This representation is then passed through two stacked bidirectional LSTM layers with 128 and 256 units, respectively, both using tanh activation and dropout regularization.

Finally, the LSTM block's output is fed into a time-distributed classification head consisting of two dense layers with 128 and 256 units, respectively, with ReLU activations, each followed by a dropout layer with a rate of 50% and 25%, respectively. The final output layer is a time-distributed dense layer with a softmax activation, producing per-frame probability predictions for every temporal instant of the input to be a wheezing event.

The model was trained for 75 epochs using the Adaptive Moment Estimation (ADAM) optimizer with a learning rate of 3e-4 and categorical cross-entropy as the loss function. Simultaneously with the training process, the model was evaluated on the validation subset at each new epoch, retaining only the set of weights with the lowest validation loss. The architecture of the AI model is represented in supplementary figure S2.

Supplementary figure S2


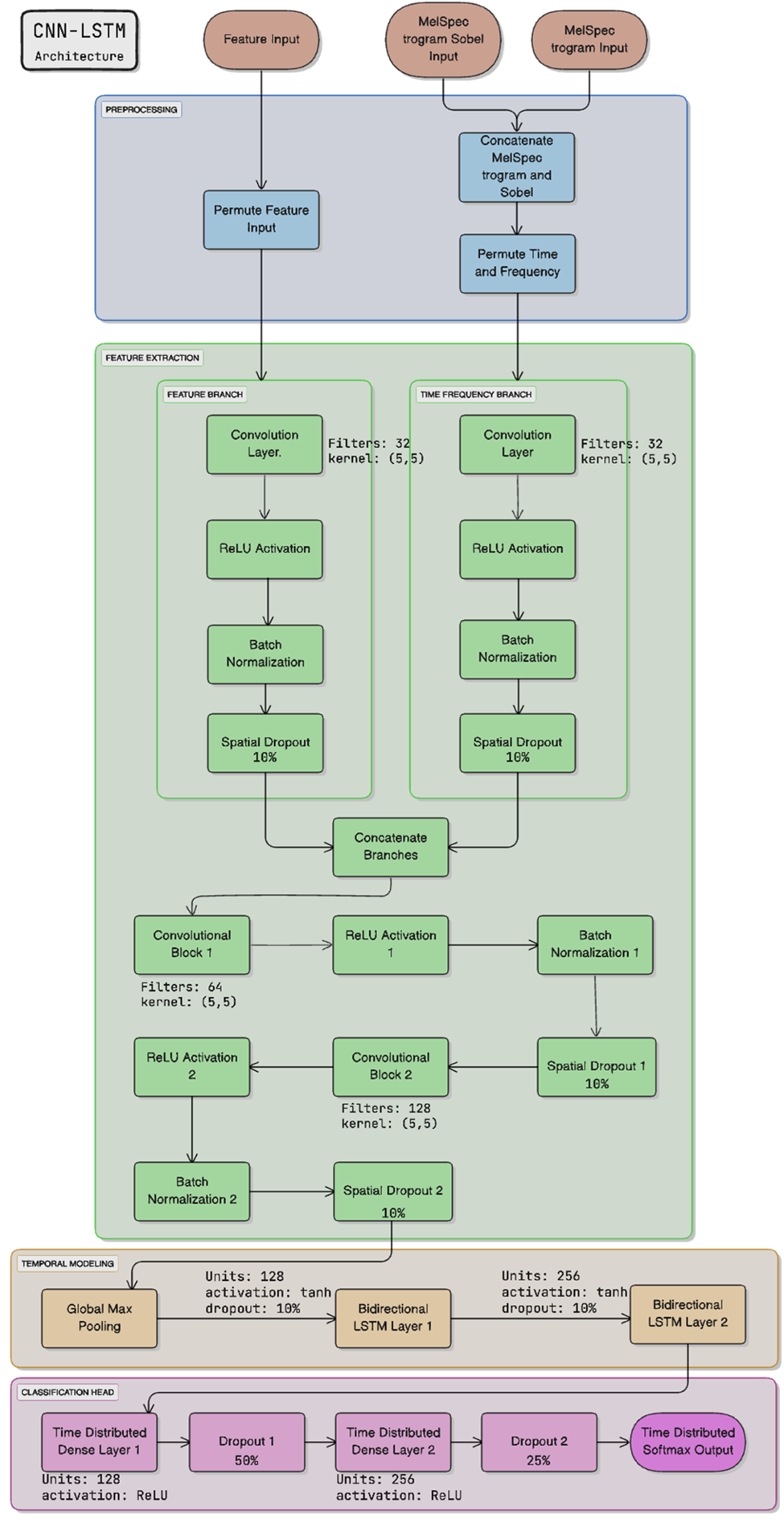


Supplementary Figure S2– Architecture of the artificial intelligent (AI) model.

# Supplementary figure S3


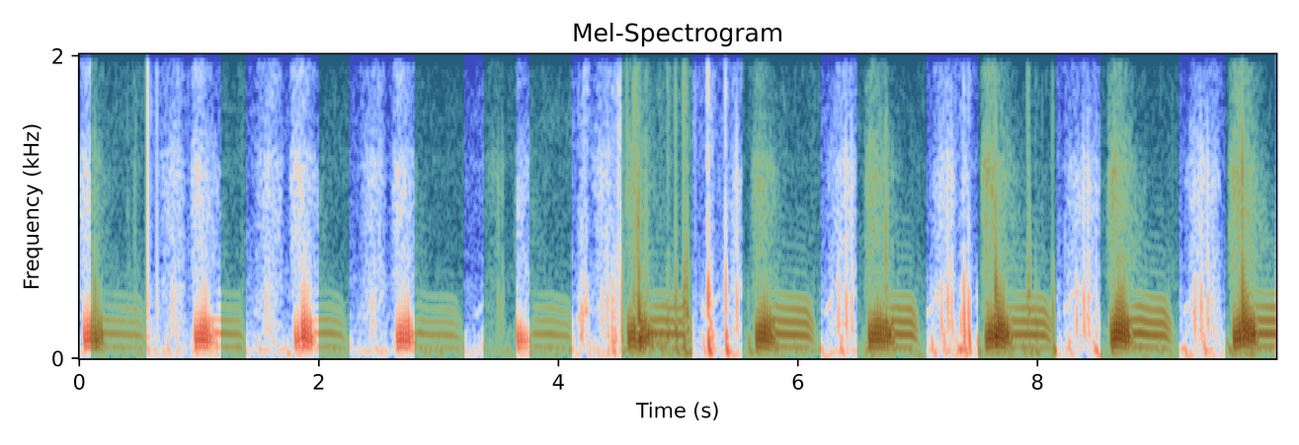
Supplementary Figure S3 – Mel-spectrogram representation of a sample recording with the output of the model (temporal onset of the detected wheezing events) highlighted in green.

# Supplementary file 3 - Inter-rater agreement on manual classification

To assess inter-rater agreement, recordings were grouped according to the annotators involved: Group 1 (HC and CJ), Group 2 (HC, CJ, and CS) and Group 3 (CJ, IPC, and MC). Regarding manual classification, 35% of recordings (n=701) were evaluated by Group 1; 30% (n=599) by Group 2 and 35% (n= 720) by Group 3. For Group 1, Cohen’s kappa was 0.80 (95%CI 0.74–0.85) for overall recording quality and 0.69 (95% CI 0.60–0.79) for the presence of wheezes. For the three-rater groups, Fleiss’ kappa ranged from 0.60 (95% CI 0.56-0.65) to 0.70 (95% CI 0.66–0.74) for recording quality and 0.58 (0.53–0.64) to 0.61 (0.55–0.66) for wheeze detection, in Groups 2 and 3, respectively.

Supplementary table S1

Characteristics of patients with respiratory sounds recordings classified as having quality (n = 209).

| **Characteristics** | | | **Total** | **Pre-school children** | | **School-aged children** | | **Adolescents** |
| --- | --- | --- | --- | --- | --- | --- | --- | --- |
| Subjects, n | | | 209 | 62 | | 41 | | 106 |
| Male, n (%) | | | 127 (60.8) | 39 (62.9) | | 24 (58.5) | | 64 (60.4) |
| Age, median [Q1–Q3] | | | 10 (4-13) | 3 (0.9-4) | | 8 (7-9) | | 13 (11-16) |
| Height, median (Q1–Q3), cm | | | 140 (109.5-158.3) | 93 (77.5-104.5) | | 128.5 (120.5-133.9) | | 158 (147.9-164.5) |
| Primary diagnosis | |  | | |  | |  | |
|  | Asthma, n (%) | | 53 (25.4) | 12 (19.4) | | 19 (46.3) | | 22 (20.8) |
|  | Cystic fibrosis, n (%) | | 15 (7.2) | 2 (3.2) | | 2 (4.9) | | 11 (10.4) |
|  | Other respiratory disease, n (%) | | 26 (12.4) | 23 (37.1) | | 0 (0) | | 3 (2.8) |
|  | No respiratory disease, n (%) | | 115 (55.1) | 25 (40.3) | | 20 (48.8) | | 70 (66.0) |

Q1, first quartile; Q3, third quartile

# Supplementary figure S4

(a)


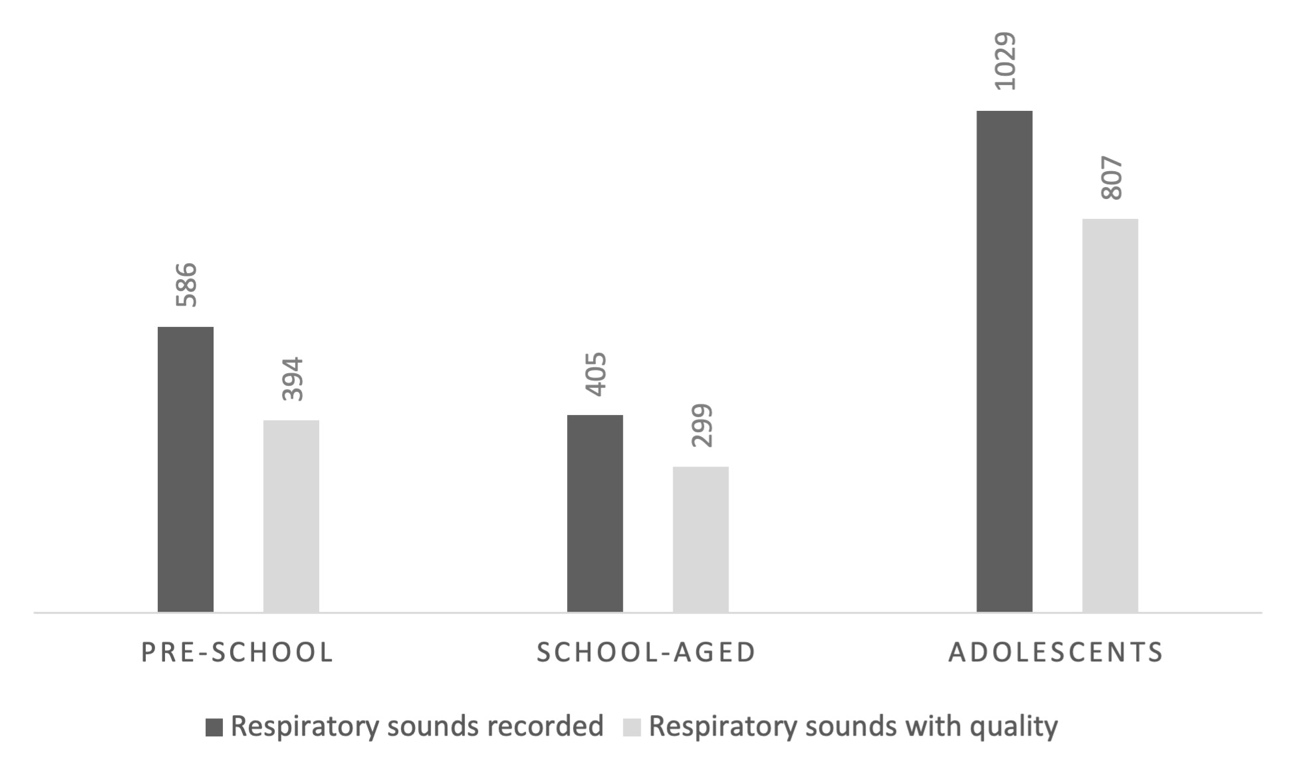


(b) **
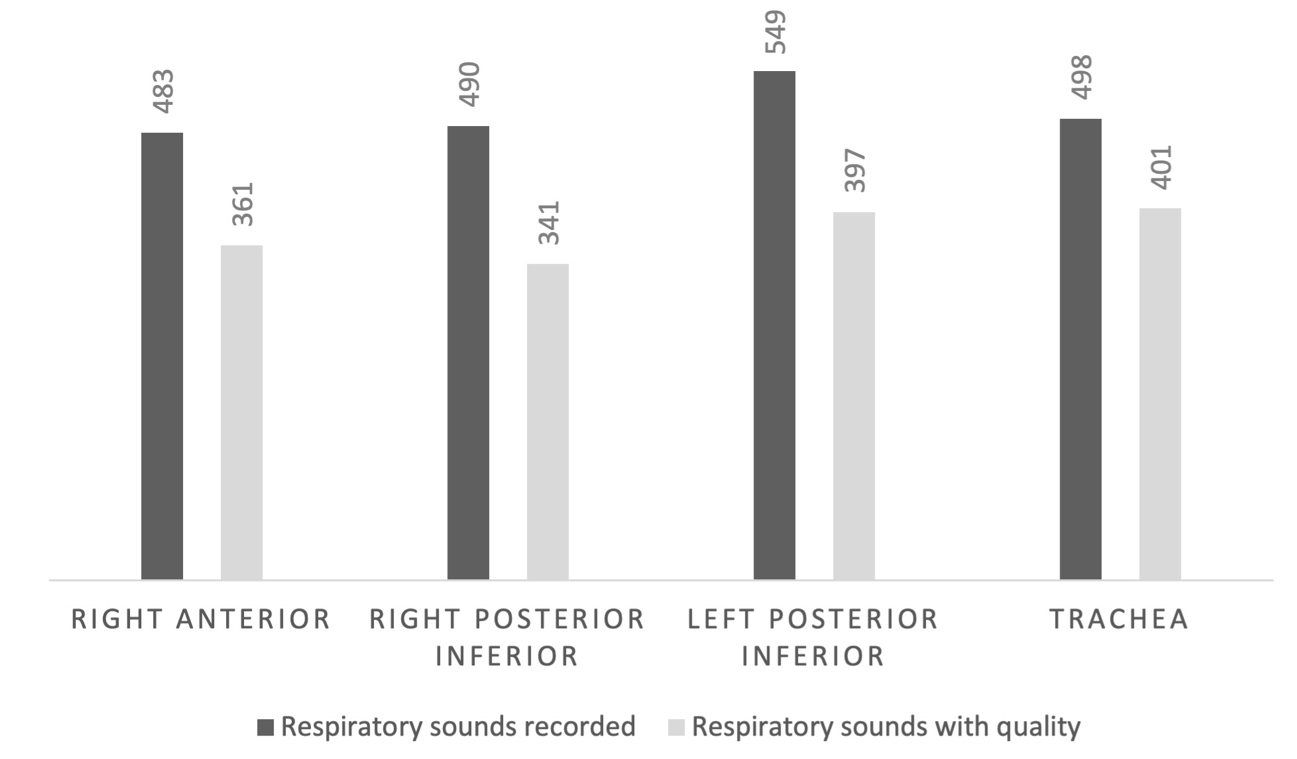
**

Supplementary Figure S4 – Graphic representation of the number of respiratory sounds recorded (n=2020) vs the number of respiratory sounds with quality (n=1500) by age group (a) and auscultation location (b).

# Supplementary figure S5


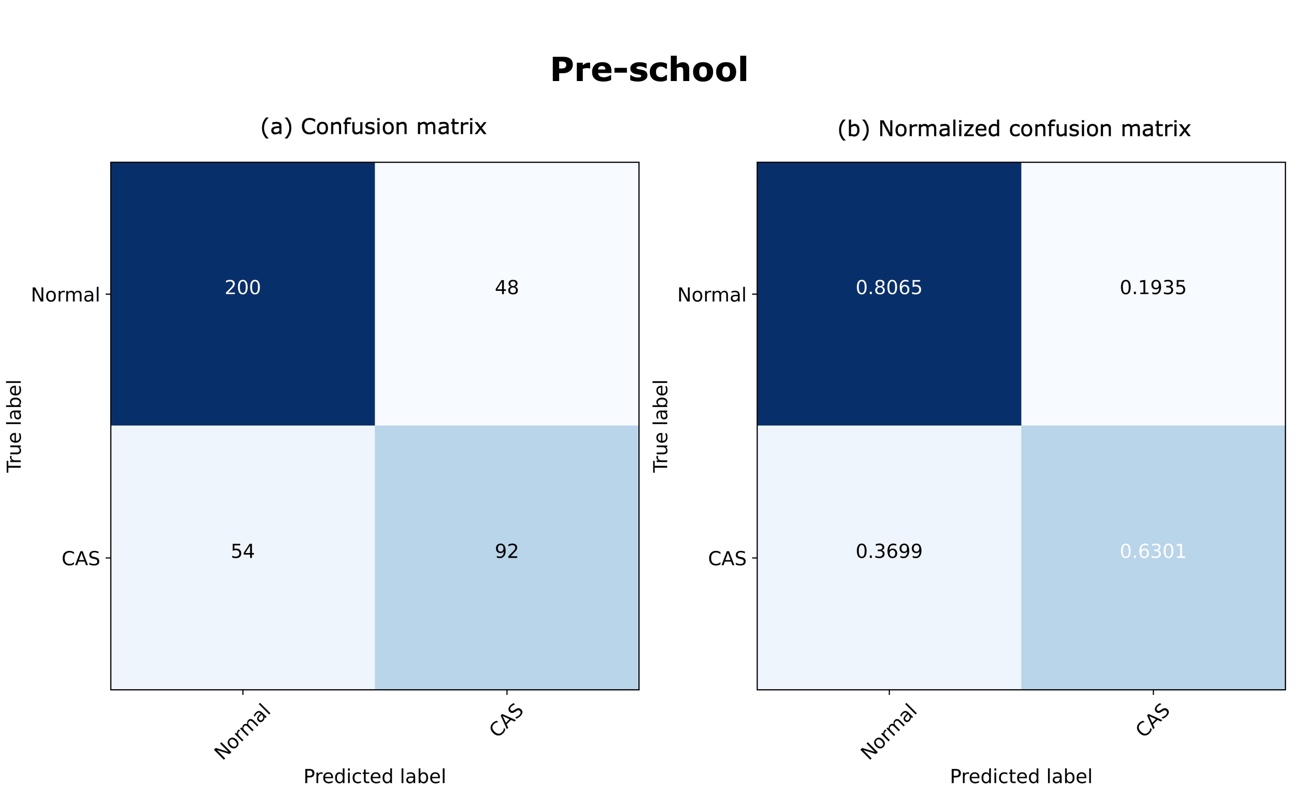


**
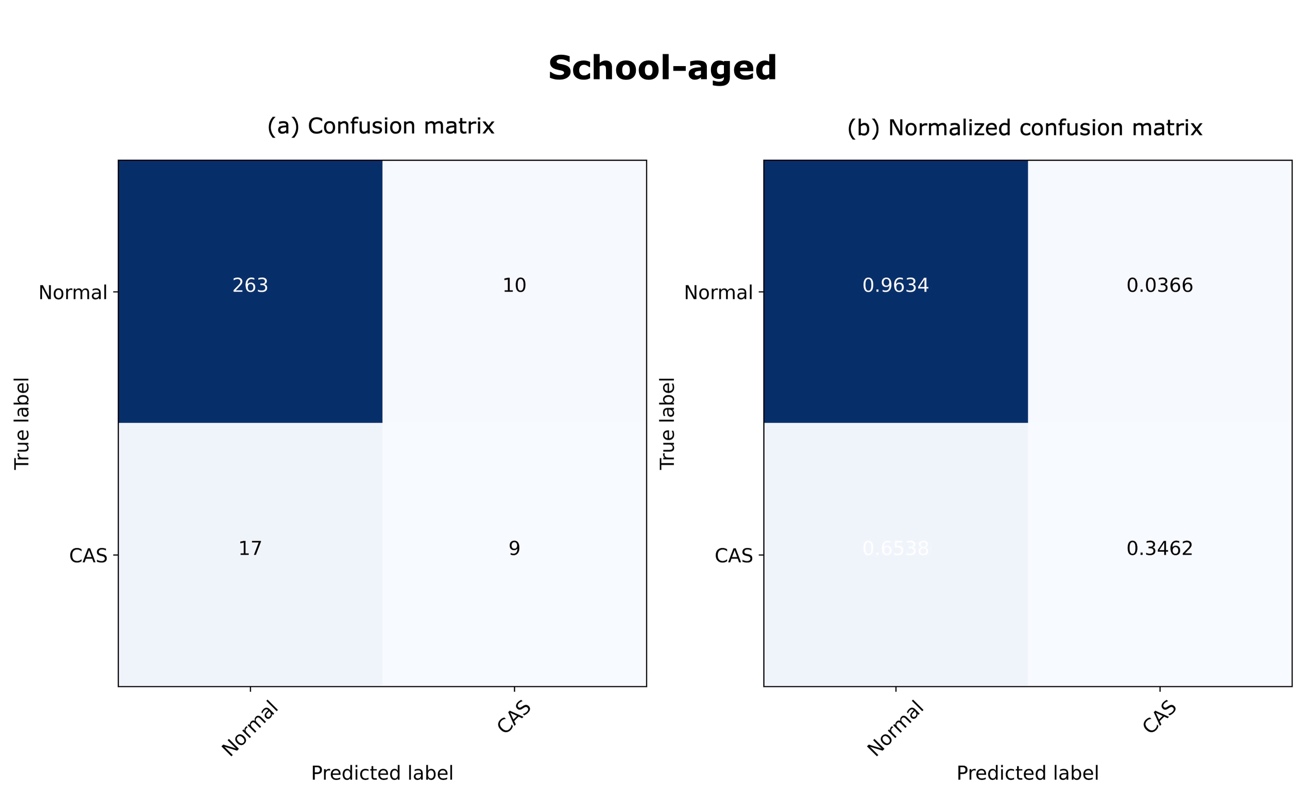
**


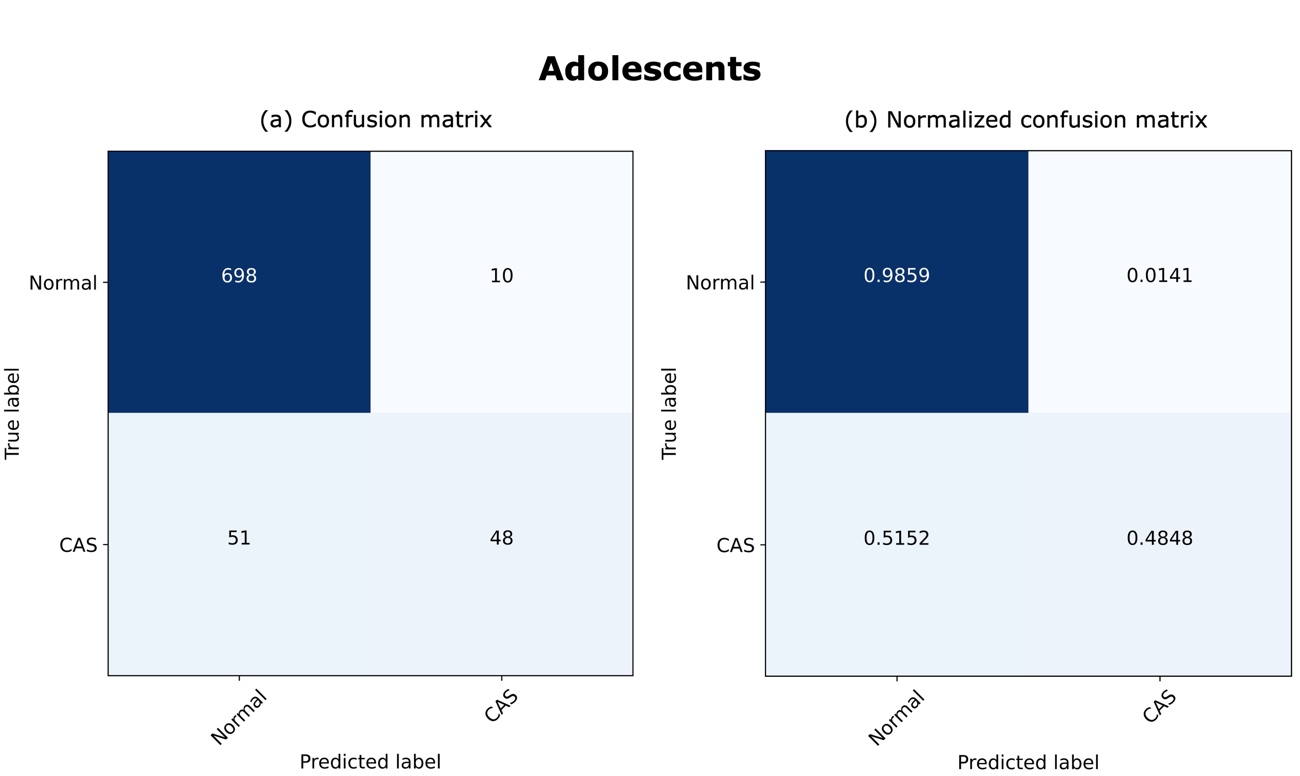


Supplementary Figure S5 – (a) Confusion matrix and (b) normalized confusion matrix of IA model performance according do age group: pre-school children, school children and adolescents (3).

# Supplementary figure S6

**
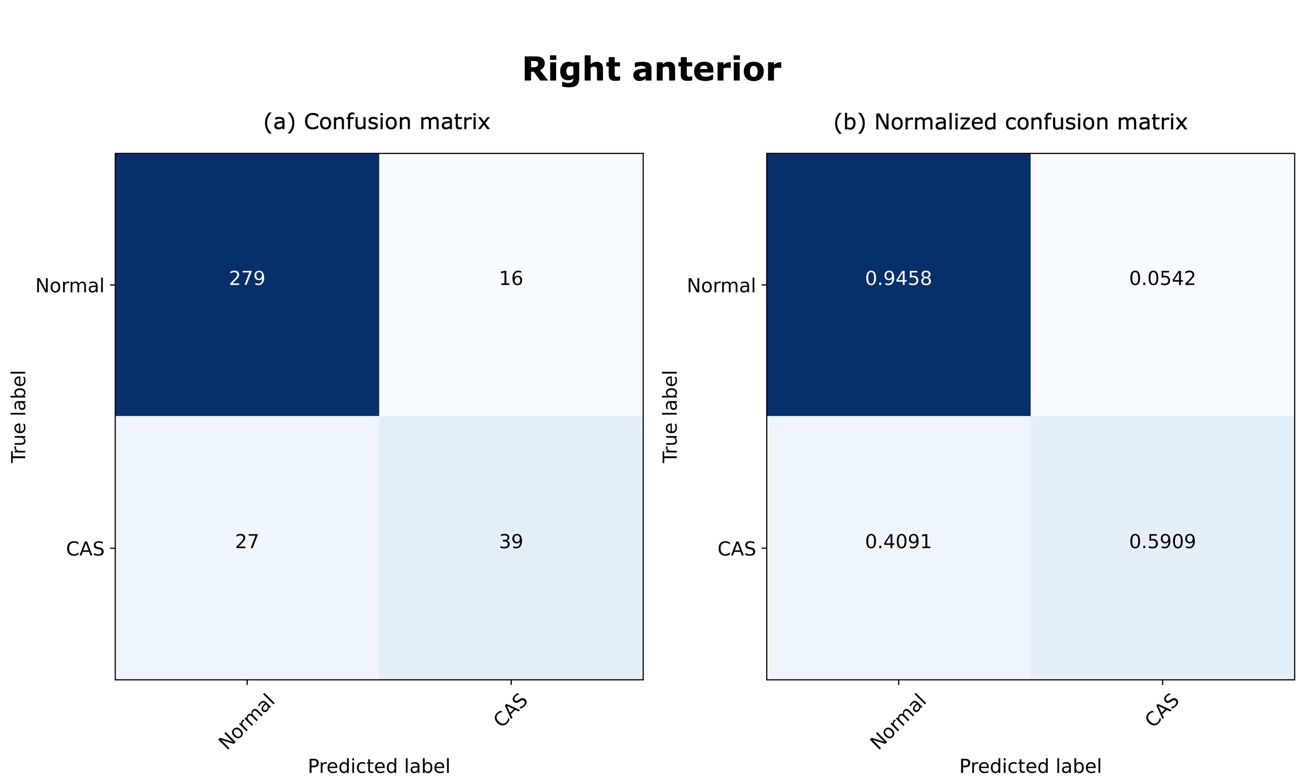

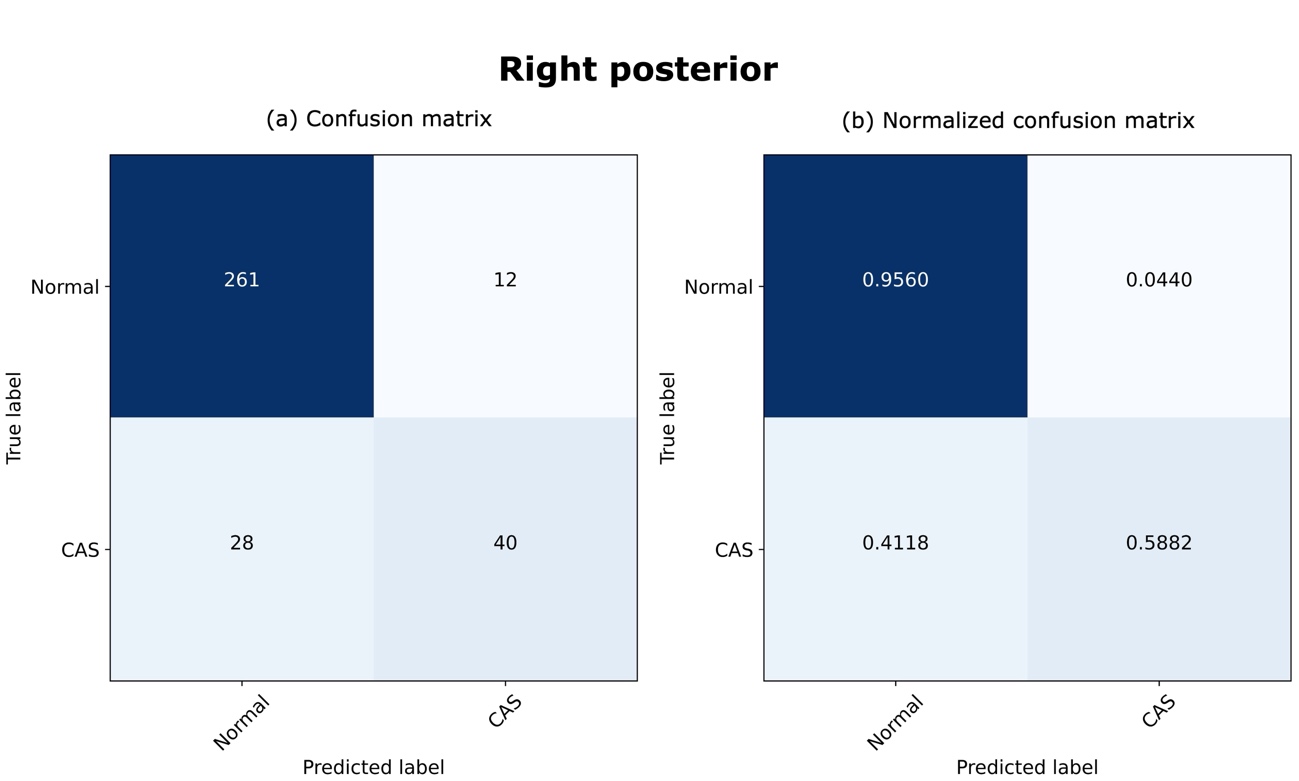
**

**
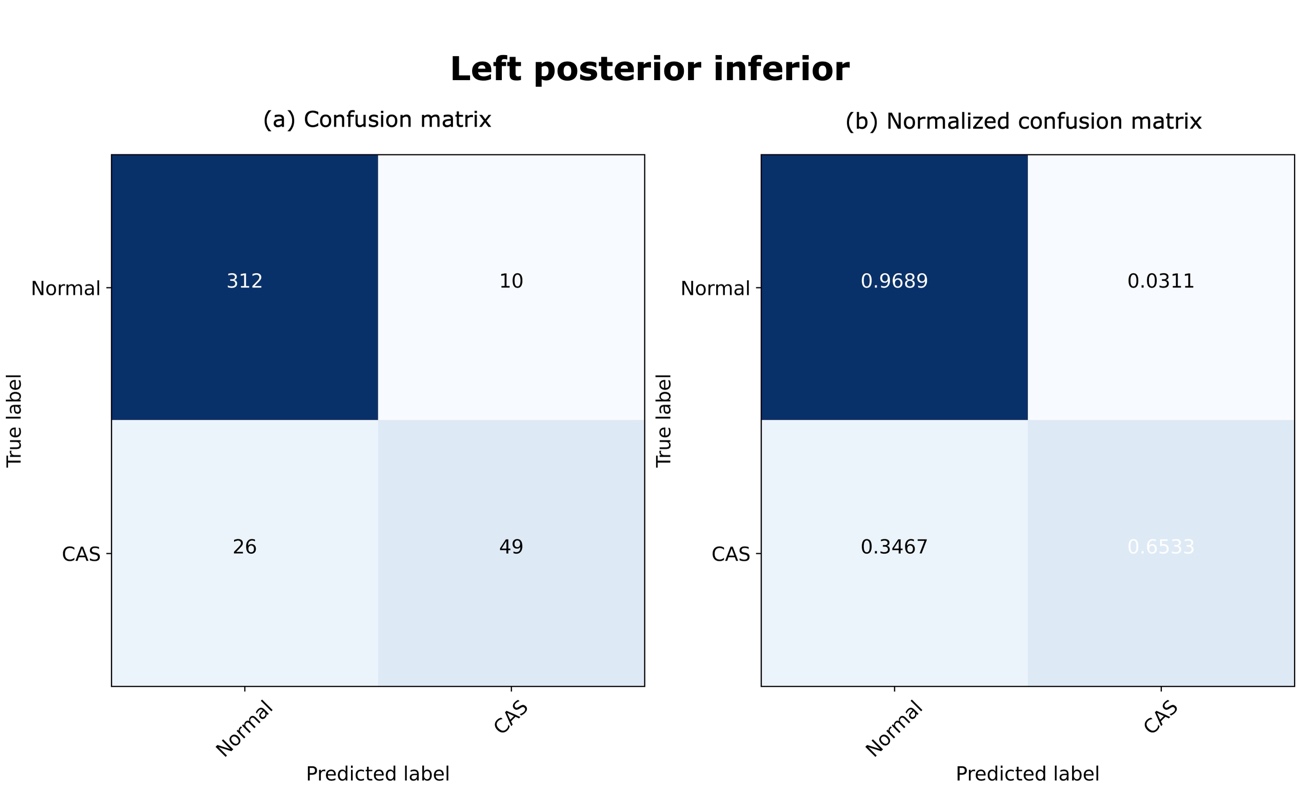

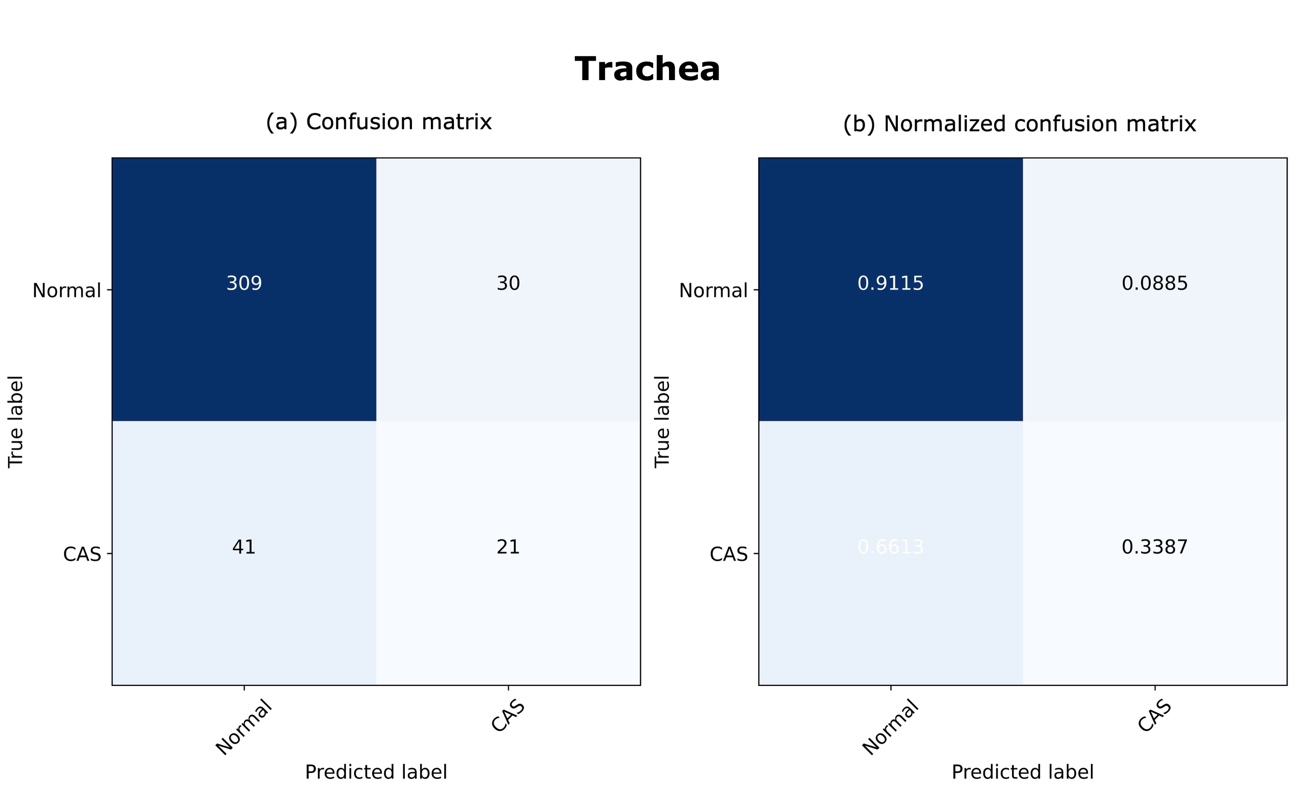
**

Supplementary Figure S6 – (a) Confusion matrix and (b) normalized confusion matrix of IA model performance according do auscultation location: right anterior inferior; right posterior inferior; left posterior inferior and trachea.

# References

1. Almeida R JC, Martinho D, et al. (2020) AIRDOC: smart mobile application for individualized support and monitoring of respiratory function and sounds of patients with chronic obstructive disease; pp. 78–88. Multi Conference on Computer Science and Information Systems MCCSIS 2020 21-25 July: Proceedings of the International Conference e-Health 2020 Macedo M, editor International Association for Development of the Information Society; 2020

2. Rossi M, Sovijärvi A, Piirilä P, Vannuccini L, Dalmasso F, Vanderschoot J (2000) Environmental and subject conditions and breathing manoeuvres for respiratory sound recordings. Eur Respir Rev 10:611-615

3. Rocha BM, Filos D, Mendes L, Serbes G, Ulukaya S, Kahya YP, Jakovljevic N, Turukalo TL, Vogiatzis IM, Perantoni E, Kaimakamis E, Natsiavas P, Oliveira A, Jácome C, Marques A, Maglaveras N, Pedro Paiva R, Chouvarda I, de Carvalho P (2019) An open access database for the evaluation of respiratory sound classification algorithms. Physiol Meas 40:035001

4. Rocha BM, Filos D, Mendes L, Vogiatzis I, Perantoni E, Kaimakamis E, Natsiavas P, Oliveira A, Jácome C, Marques A, Paiva RP, Chouvarda I, Carvalho P, Maglaveras N (2018) Α Respiratory Sound Database for the Development of Automated Classification. In: Maglaveras N, Chouvarda I, de Carvalho P (eds) Precision Medicine Powered by pHealth and Connected Health. Springer Singapore, Singapore, pp 33-37

5. Hsu F-S, Huang S-R, Huang C-W, Cheng Y-R, Chen C-C, Hsiao J, Chen C-W, Lai F (2022) A Progressively Expanded Database for Automated Lung Sound Analysis: An Update. Applied Sciences 12:7623

6. Zhang Q, Zhang J, Yuan J, Huang H, Zhang Y, Zhang B, Lv G, Lin S, Wang N, Liu X, Tang M, Wang Y, Ma H, Liu L, Yuan S, Zhou H, Zhao J, Li Y, Yin Y, Zhao L, Wang G, Lian Y (2022) SPRSound: Open-Source SJTU Paediatric Respiratory Sound Database. IEEE Transactions on Biomedical Circuits and Systems 16:867-881

7. Pessoa D, Machado Rocha B, de Carvalho P, Paiva RP (2022) Chapter 5 - Automated respiratory sound analysis. In: Paiva RP, Carvalho Pd, Kilintzis V (eds) Wearable Sensing and Intelligent Data Analysis for Respiratory Management. Academic Press, pp 123-168

8. Vincent O, & Folorunso, O. (2009) A Descriptive Algorithm for Sobel Image Edge Detection. .Paper presented at InSITE 2009: Informing Science + IT Education Conference.

9. Pessoa D, Rocha BM, Gomes M, Rodrigues G, Petmezas G, Cheimariotis G-A, Maglaveras N, Marques A, Frerichs I, de Carvalho P, Paiva RP (2024) Ensemble deep learning model for dimensionless respiratory airflow estimation using respiratory sound. Biomedical Signal Processing and Control 87:105451
